# POPX2 is a novel LATS phosphatase that regulates the Hippo pathway

**DOI:** 10.18632/oncotarget.26689

**Published:** 2019-02-19

**Authors:** Muhammad Bakhait Rahmat, Songjing Zhang, Cheng-Gee Koh

**Affiliations:** ^1^ Interdisciplinary Graduate School, Nanyang Technological University, Singapore; ^2^ School of Biological Sciences, Nanyang Technological University, Singapore

**Keywords:** Hippo pathway, POPX2 phosphatase, LATS1 kinase, YAP/TAZ, anchorage-independent growth

## Abstract

The Hippo pathway regulates cell proliferation, survival, apoptosis and differentiation. During carcinogenesis, members of the Hippo pathway are mutated to avoid anoikis and promote anchorage independent growth. Although many regulators of the Hippo pathway have been reported, negative regulators of the hippo kinases are not well studied. Through an interactome screen, we found that POPX2 phosphatase interacts with several of the Hippo pathway core kinases, including LATS1 which is the direct kinase regulating the transcription co-activators, YAP and TAZ. Phosphorylated YAP/TAZ are retained in the cytoplasm and prevented from translocation into the nucleus to activate transcription of target genes. We found that POPX2 could dephosphorylate LATS1 on Threonine-1079, leading to inactivation of LATS1 kinase. As a result, YAP/TAZ are not phosphorylated and are able to translocate into the nucleus to activate target genes involved in cell proliferation. Furthermore, POPX2 knock-out using CRISPR in the highly metastatic MDA-MB-231 breast cancer cells results in decreased cell proliferation and impairment of anchorage independent growth. We propose that POPX2 act as a suppressor of the Hippo pathway through LATS1 dephosphorylation and inactivation.

## INTRODUCTION

During carcinogenesis, tumor cells acquire mutations that enable them to grow uncontrollably. These feats are achieved through the activation of select developmental and signaling pathways. The Hippo pathway plays major roles in regulating organ size and is relevant to tissue regeneration [1]. It has also been implicated in cancer development [2]. The Hippo pathway constitutes a cascade of kinases that phosphorylate one another leading to the phosphorylation and inactivation of YAP and TAZ transcription co-activators. The Hippo pathway kinase cassette is made up of the MST1/2 kinases and the LATS1/2 kinases. MST1/2 phosphorylate and activate LATS1/2. Recently the NDR1/2 kinases are found to be new members of the Hippo pathway, which can phosphorylate YAP [3]. Other members of the Hippo pathway include MOB1 which serves as an adaptor for the recruitment and regulation of NDR1 and LATS1/2. Phosphatases such as PP2A and PP1A have been implicated in the regulation of the Hippo pathway by dephosphorylating and inactivating the kinases in the Hippo pathway and YAP/TAZ [4–7].

YAP and TAZ are related transcription co-activators and orthologs of the *Drosophila* Yorkie. They can shuttle between the cytoplasm and nucleus to interact with transcription factors such as Tea domain family members (TEAD) to induce gene expression [8]. YAP/TAZ are phosphorylated by LATS1/2 and NDR1/2. While in the non-phosphorylated state, active YAP/TAZ associate with transcription factors to promote cell proliferation, differentiation and survival. Phosphorylated YAP/TAZ are retained in the cytoplasm and may be targeted for degradation [9]. Both YAP/TAZ are established oncogenes in various cancers [10]. Elevated levels of YAP/TAZ have been reported in many cancer types. Prominently, TAZ abundance is elevated in invasive breast cancer cell lines, where it is observed that high TAZ expression confers breast cancer cells with cancer stem cells traits and induces epithelial-mesenchymal transition (EMT) [11].

Partner of PIX 2 (POPX2/CaMKP/PPM1F) phosphatase belongs to the PP2C family of serine/threonine protein phosphatase. Its expression is ubiquitous and is found in most human tissues. Currently, four POPX2 substrates have been reported; they are p21-activated kinase (PAK) [12], calcium-calmodulin kinase II (CaMKII) [13], KIF3A kinesin motor protein [14] and TGF-β activated kinase (TAK1) [15]. POPX2 also interacts with the formin protein mDia1 and modulates RhoA pathways [16]. Previously we have reported that the expression of POPX2 correlates with invasiveness of breast cancer cell lines [17]. The phosphatase is also implicated in the regulation of stress fibers, focal adhesions, cell migration, polarity and apoptosis [15, 18–20]. To uncover additional pathways regulated by POPX2, we performed immunoprecipitation of overexpressed tagged-POPX2 and identified two proteins belonging to the Hippo pathway within the population of POPX2 associated proteins using mass spectrometry (Weng and Koh, unpublished data). The two proteins identified were NDR1 and MOB1, components of the Hippo core kinase cassette. Therefore, we investigated further to determine if POPX2 has a role in the regulation of the Hippo kinases.

Here, we report that POPX2 functions as a LATS1 phosphatase. We found that POPX2 could dephosphorylate LATS1 on its activation site Threonine-1079 resulting in inactivation of LATS1. As a result, TAZ remains non-phosphorylated. Loss of POPX2 resulted in less cytoplasmic and nuclear TAZ. Furthermore, knocking out POPX2 in MDA-MB-231 cells led to reduced cell proliferation and lower growth in soft agar assays. Our study has uncovered POPX2 as a novel negative regulator of the Hippo pathway.

## RESULTS

### POPX2 interacts with multiple proteins in the Hippo pathway

In a pulldown/mass-spectrometry interactome screen using Flag-tagged POPX2 as a bait, we have identified TAK1 and other proteins as POPX2 binding proteins [15]. Amongst the list of possible POXP2 interactors, we also found NDR1 and MOB1 which are components of the Hippo core kinase cassette. This discovery led us to investigate further to determine if POPX2 has a role in the regulation of Hippo kinases.

**Figure 1:**
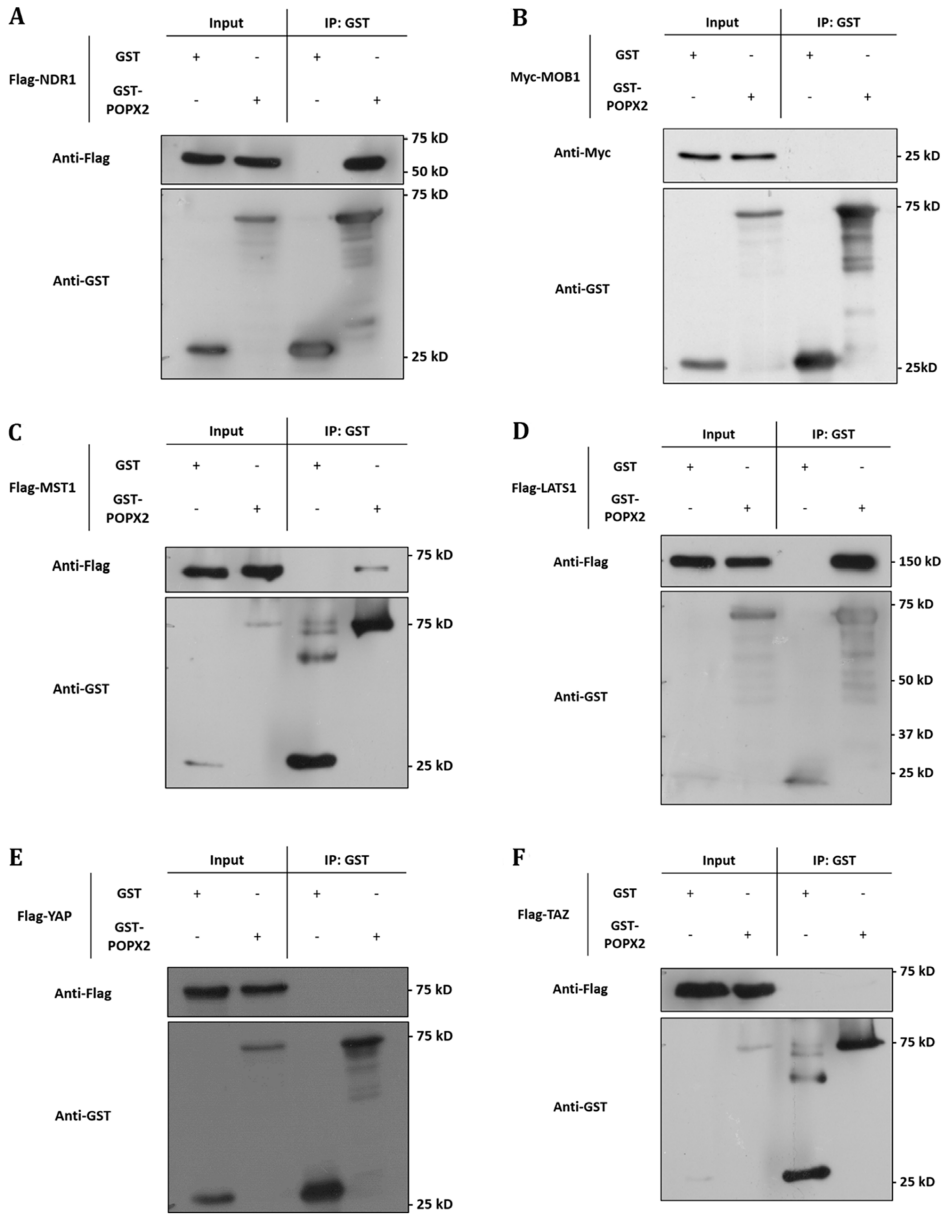
POPX2 selectively binds to members of the Hippo pathway Lysates from HEK293 cells expressing GST-vector or GST-POPX2 together with (**A**) Flag-NDR1, (**B**) Myc-MOB1, (**C**) Flag-MST1, (**D**) Flag-LATS1, (**E**) Flag-YAP, and (**F**) Flag-TAZ were subjected to immunoprecipitation with glutathione sepharose beads and western analysis. Proteins associated with GST-POPX2 were detected by Western blotting (WB) with either anti-Flag or anti-Myc antibodies.

**Figure 2:**
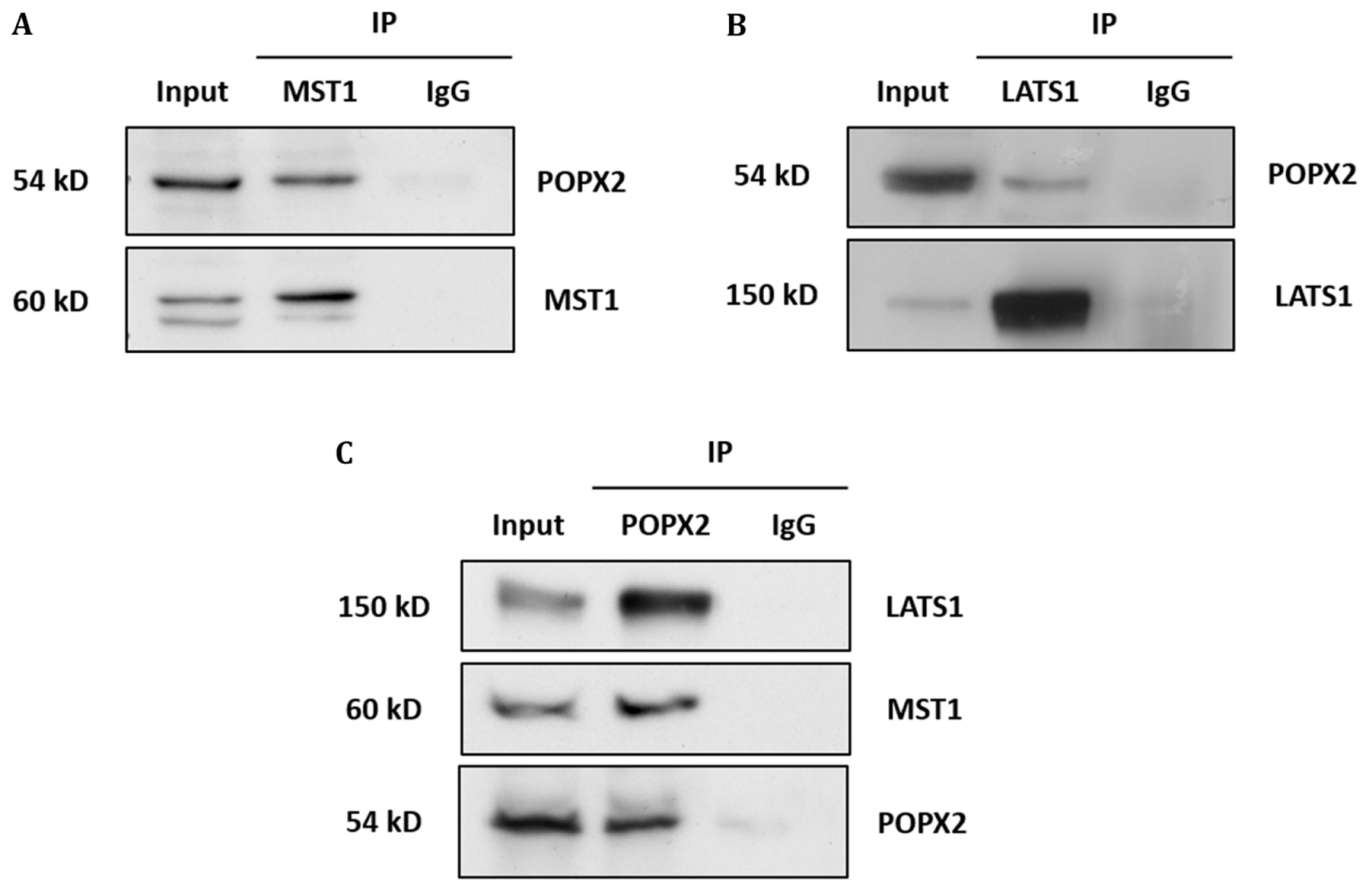
Endogenous interaction of POPX2 and Hippo kinases (**A**) Endogenous MST1 from HEK293 cells was immunoprecipitated. The IP complexes were separated by SDS PAGE, followed by Western analysis using POPX2 antibody. The Western blot reveals endogenous MST1-POPX2 interaction. (**B**) Similar experiment as in (A) was performed using LATS1 antibody for immunoprecipitation. The Western blot reveals endogenous LATS1-POPX2 interaction. (**C**) Reciprocal endogenous POPX2 immunoprecipitation followed by Western analysis using MST1 and LATS1 antibodies reveal endogenous POPX2-MST1 and POPX2-LATS1 interactions. Endogenous immunoprecipitation experiments are representative of 2 independent experiments; representative images were shown.

To validate the interactions, we performed co-immunoprecipitation of GST-tagged POPX2 with Flag-tagged NDR1 or MOB1 (Figure 1A and 1B). We found that NDR1 but not MOB1 could be detected in the pulldown complex of POPX2. We next investigated whether POPX2 also formed complexes with other members of the Hippo pathway by co-immunoprecipitation assays (Figure 1C–1F). We found that in addition to NDR1, MST1 (Figure 1C) and LATS1 (Figure 1D) could be detected in POPX2 pulldown but not YAP nor TAZ (Figure 1E and 1F). We next performed immunoprecipitation to detect endogenous POPX2-MST1 and POPX2-LATS1 complexes in the cells. We could detect endogenous POPX2 amongst the proteins which co-precipitated with MST1 or LATS1 and *vice versa* in immunoprecipitation experiments (Figure 2). These observations suggest that physical interactions between POPX2 and the Hippo pathway members are selective and restricted to the core kinases.

### POPX2 dephosphorylates LATS1 but not MST1

Both MST1 and LATS1 are activated by phosphorylation. MST1 has been reported to undergo autophosphorylation and MST1-Thr183 phosphorylation is essential for its kinase activity [21]. Similarly, Ser909 and Thr1079 of LATS1 are identified to be phosphorylated by MST2 and essential for LATS1 kinase activity [22]. Since POPX2 interacts with both MST1 and LATS1, we hypothesize that both kinases might be substrates of POPX2. POPX2 might negatively regulate the activities of MST1 and LATS1 through dephosphorylation.

**Figure 3:**
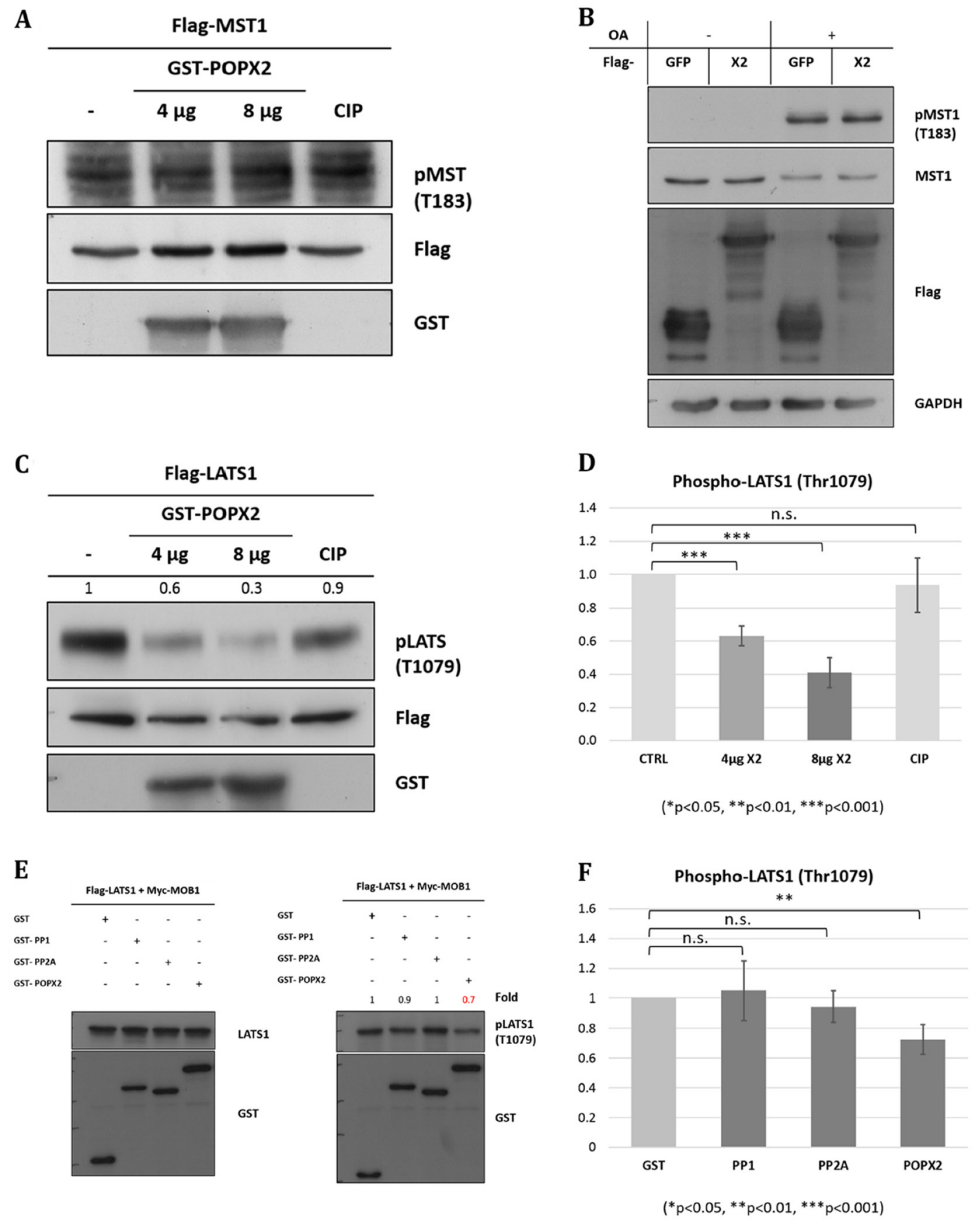
POPX2 dephosphorylates LATS1 but not MST1 (**A**) *In vitro* phosphatase assay. Bacterially expressed POPX2 (4 µg and 8 µg) was incubated with phospho-Flag-MST1 for 45 mins at 30°C. The reaction mixtures were subjected to SDS-PAGE and Western analysis using phospho-MST1-Thr183 antibodies. Calf-intestinal phosphatase (CIP) was used as a negative control. (**B**) HEK293 cells were transfected with GFP-POPX2 or GFP-Flag vector constructs. The cells were treated with okadaic acid (1 µM) for 1 hr to inhibit PP2A and induce MST1 autophosphorylation. Cell lysates were separated on SDS-PAGE and subjected to Western analysis to detect phospho-MST1-Thr183. (**C**) *In vitro* phosphatase assay. Bacterially expressed POPX2 was incubated with phospho-Flag-LATS1 instead of Flag-MST1 as in (A). The reaction mixtures were subjected to SDS-PAGE and Western analysis using phospho-LATS1-Thr1079 antibodies. Calf-intestinal phosphatase (CIP) was used as a negative control. CIP can remove phosphate group from phospho-tyrosine, serine and threonine, but with a preference for phospho-tyrosine. In our experiment, CIP was used as a negative control because when CIP was used in similar experimental condition as POPX2 for phosphatase assays, it was found not as efficient as POXP2 in dephosphorylating LATS1. (**D**) Densitometry quantification of (C) from three independent experiments. POPX2 dephosphorylates LATS1 in a dose dependent manner. (**E**) HEK293 cells were transfected with Flag-LATS1 + Myc-MOB1 together with GST-vector, GST-PP1, GST-PP2A or GST-POPX2. Cell lysates were harvested and separated by SDS-PAGE and subjected to Western analysis. Equal amounts of total protein lysates were loaded into each well of the gel. Total LATS1 (left panel) and pLATS1 (right panel) were probed on separate membranes. (**F**) Densitometry quantification of phospho-LATS1. Three independent experiments were performed. Error bars represent standard deviation. Student *t*-test was performed to determine statistical significances.

To test our hypothesis, bacterially purified GST-POPX2 was mixed with phosphorylated Flag-MST1 in an *in vitro* phosphatase assay (Figure 3A). To obtain phosphorylated Flag-MST1, HEK293 cells transfected with Flag-MST1 were treated with Calyculin A (50 nM, 1 hr) to stimulate Thr183 phosphorylation before immunoprecipitation using anti-Flag antibodies (Supplementary Figure 1A). Inhibition of PP2A can lead to MST1 auto-phosphorylation and activation [23]. We also attempted to check for dephosphorylation of MST1-Thr183 *in vivo* by transfecting POPX2 cDNA construct into HEK293 cells and detect for phospho-MST1-Thr183 levels in the cell lysates (Figure 3B). In this experiment, we used Okadaic acid (1 µM) instead of Calyculin A for inhibition of PP2A [24] to induce MST1 auto-phosphorylation. We observed that there was increase in phospho-MST1-Thr183 levels when cells are treated with Okadaic acid. However, we failed to detect dephosphorylation of MST1-Thr183 by POPX2 in *in vitro* phosphatase assays and overexpression situations (Figure 3A and 3B).

We next investigated whether POPX2 is capable of dephosphorylating LATS1-Thr1079. Bacterially purified GST-POPX2 was mixed with phosphorylated Flag-LATS1 in an *in vitro* phosphatase assay (Figure 3C). To obtain phosphorylated Flag-LATS1, Flag-LATS1 was co-transfected with Myc-MOB1 into HEK293 and then treated with Calyculin A (50 nM, 1 hr) to further enhance Thr1079 phosphorylation (Supplementary Figure 1B), thereafter phospho-LATS1 was pulldown using Anti-Flag M2 mouse antibody. We observed that POXP2 was able to dephosphorylate LATS1 with high efficiency and in a dose dependent manner in *in vitro* phosphastase assays (Figure 3C and 3D). Calf intestinal phosphatase (CIP) was not effective in dephosphorylating LATS1 under similar experimental conditions. Overexpression of POPX2 resulted in reduced phospho-LATS1 (Supplementary Figure 1B). Consistent with this, we observed that unlike PP2A and PP1, overexpression of POPX2 resulted in reduction of phospho-LATS1-Thr1079 levels in HEK293 cells (Figure 3E and 3F) in a significant manner. We have earlier confirmed that co-transfection with MOB1 is sufficient to activate LATS1 (Supplementary Figure 2). Our experimental results suggest that LATS1 but not MST1 is a substrate of POPX2.

### Loss of POPX2 affects MDA-MB-231 morphology a well as TAZ and vimentin protein levels

After establishing that POPX2 indeed regulates LATS1 phosphorylation (Figure 3C–3F), we investigate further the effects of POXP2 on the Hippo pathway. More specifically, we wish to determine if the levels of POPX2 can affect the activity of YAP/TAZ. To do so, we compare MDA-MB-231 wildtype cells with POPX2 CRISPR knock-out MDA-MB-231 cells [henceforth known as X2KO cells (Zhang and Koh, unpublished cell line)].

**Figure 4:**
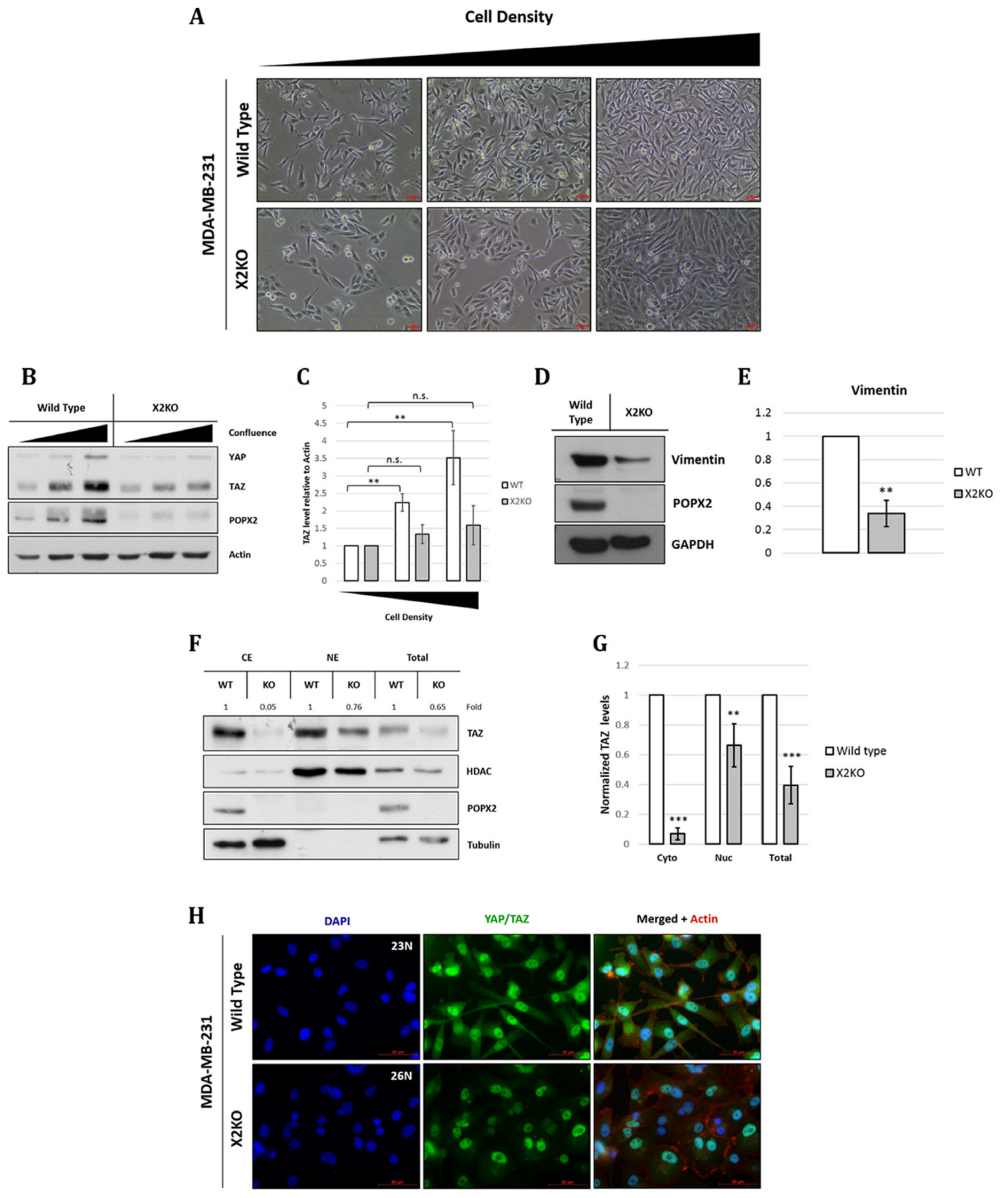
Loss of POPX2 affects MDA-MB-231 morphology a well as TAZ and vimentin protein levels (**A**) Brightfield images of MDA-MB-231 wild type and X2KO cells at increasing cell density. Scale bar: 50 µm. (**B**) Cell lysates at different cell densities were separated by SDS-PAGE, and subjected to western analysis. YAP/TAZ protein levels at different cell density were probed using antibodies against YAP/TAZ. (**C**) Densitometry quantification of TAZ protein levels relative to actin at different cell density. Three independent experiments were performed. Error bars represent standard deviation. Student *t*-test was performed to determine statistical significance. ^**^p < 0.01, ^***^p < 0.001 (**D**) Cell lysates were separated by SDS-PAGE, subjected to western analysis using vimentin antibody. (**E**) Densitometry quantification of vimentin protein levels relative to GAPDH. Three independent experiments were performed. Error bars represent standard deviation. Student *t*-test was performed to determine statistical significance. ^**^p < 0.01 (**F**) Loss of POPX2 affects cytosolic and nuclear TAZ protein levels. MDA-MB-231 wild type and X2KO cells were lysed and fractionated into cytoplasmic, nuclear fractions and total extract. Extracts were then separated by SDS-PAGE and subjected to western analysis. (**G**) Densitometry quantification of (**F**). Three independent experiments were performed. Error bars represent standard deviation. Student *t*-test was performed to determine statistical significance. (**H**) MDA-MB-231 wild type and X2KO cells grown to high cell density were immunostained with YAP/TAZ antibody. Scale bar: 50 µm.

In non-transformed cells, YAP/TAZ activity is known to change with cell density, where YAP/TAZ activity is inversely correlated with cell density [25]. At high cell density, YAP/TAZ are localized to the cytoplasm; whereas at low cell density, YAP/TAZ translocate to the nucleus to induce cell proliferation. YAP and TAZ function as oncogenes in many cancer types. Changes to the expression of YAP/TAZ with respect to cell density have not been studied in transformed cells such as MDA-MB-231. Thus, we studied both the morphological and biochemical changes in control MDA-MB-231 and X2KO cells (Figure 4) in different cell densities. We observed that X2KO cells displayed more cell-cell contact, and overall a more epithelial and less spindle shaped (mesenchymal) morphology (Figure 4A) compared with control MDA-MB-231 cells. Unlike non-transformed cells, the levels of YAP and TAZ increased with increasing cell density in control MDA-MB-231 cells (Figure 4B and 4C). This may suggest that transformed cell types might not be able to regulate the Hippo pathway. However, the overall increment of YAP and TAZ levels were dampened in X2KO cells (Figure 4B and 4C). There appear to be much less YAP than TAZ in MDA-MB-231 cells. To verify the possibility of mesenchymal-epithelial transition (MET), we probed for the mesenchymal marker vimentin and observed that vimentin level was reduced in X2KO cells (Figure 4D and 4E).

To further decipher if POPX2 affects TAZ’s localization in the cytoplasm and nucleus, we next performed fractionation experiment to determine the levels of TAZ in the different cell fractions. The overall levels of TAZ were much less in X2KO cells. In particular, there appeared to be very little TAZ in the cytoplasm of X2KO cells (Figure 4F and 4G). The majority of TAZ in X2KO cells are localized to the nuclei. Nevertheless, the levels of nuclear TAZ in X2KO cells were estimated to be 0.6 times that of control cells. Consistent with the western analysis, immuno-cell staining showed that overall cytoplasmic, nuclear and total TAZ protein levels in X2KO cells were lower compared to control MDA-MB-231 cells (Figure 4H).

### Loss of POPX2 affects TAZ target gene expressions and growth

**Figure 5:**
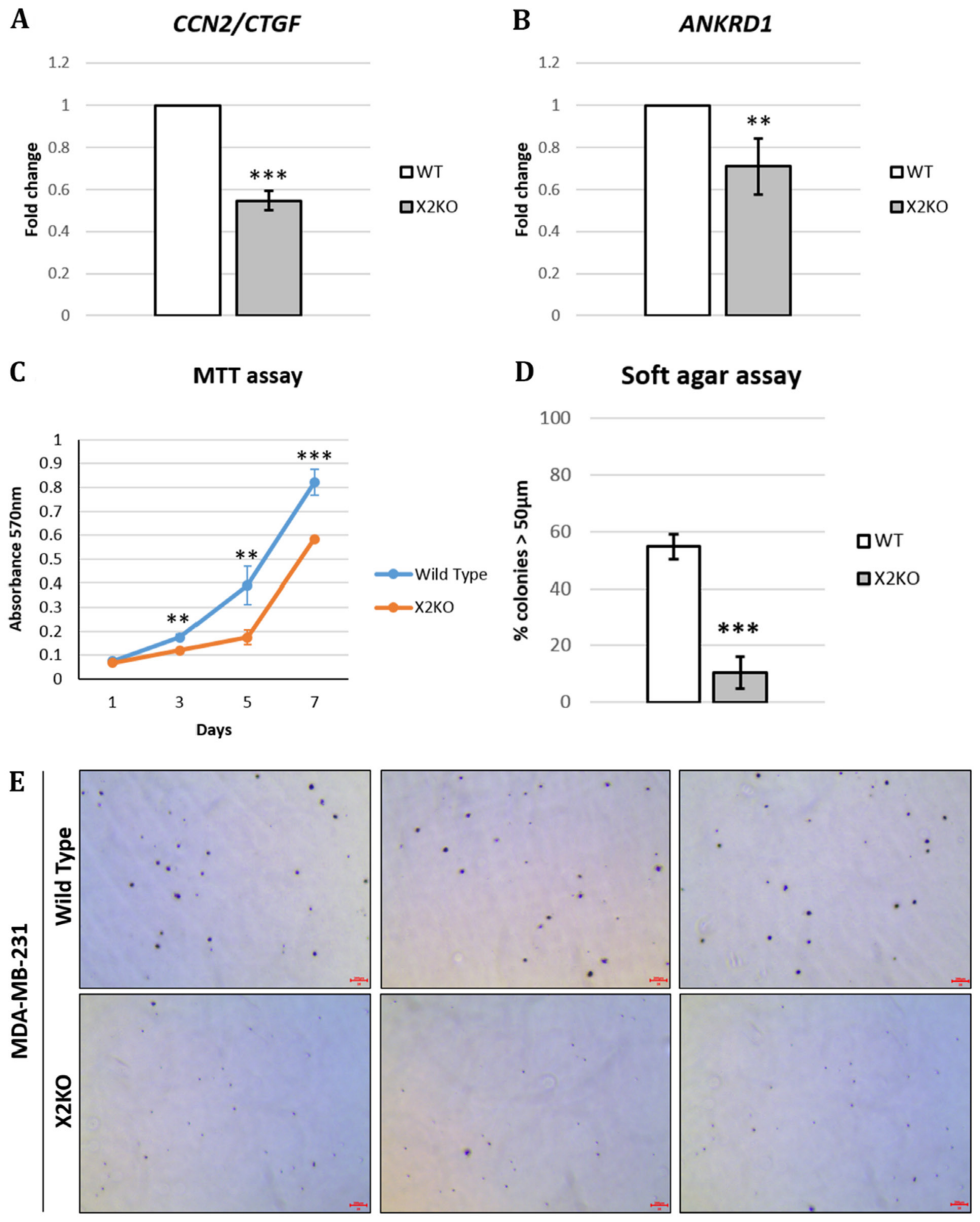
Loss of POPX2 reduces TAZ target gene expression and growth MDA-MB-231 wild type and X2KO cells were grown to high cell density. Cells were harvested and the RNA collected were converted to cDNA and subjected to RT-PCR to quantify the amount of (**A**) *CTGF* and (**B**) *ANKRD1* transcript levels. (**C**) Cell proliferation were quantified using MTT assays on Day 1, 3, 5 and 7. Three independent experiments were performed. Error bars represent standard deviation. Student *t*-test was performed to determine statistical significance. ^**^p < 0.01, ^***^p < 0.001. (**D**) Cells were grown in 3D soft agar assay for up to 28 days. The numbers of total colonies and colonies with diameter more than 50 μm were counted. Three independent experiments were performed. Error bars represent standard deviation. Student *t*-test was performed to determine statistical significance. (**E**) Representative bright field images of crystal violet stained cell colonies in soft agar assay. Scale bar: 200 µm.

Following our observation that the loss of POPX2 resulted in lower TAZ protein levels (Figure 4F–4H), we hypothesized that lower overall TAZ protein levels could translate to lower expression of TAZ target genes. Therefore, we determined the expression of TAZ target genes, connective tissue growth factor (*CTGF*) and ankaryn repeat domain 1 (*ANKRD1*), using RT-PCR. Total RNA was extracted from control MDA-MB-231 and X2KO cells. RT-PCR was performed and the fold change was computed using ∆∆CT method (Figure 5A and 5B). Consistent with lower overall and nuclear TAZ levels, we observed that X2KO cells displayed lower TAZ target gene expression. These observations suggest lower TAZ activity in X2KO cells. We further examined the effects of lower TAZ levels on cell proliferation and anchorage independent growth using MTT and soft agar assays, respectively (Figure 5C–5E). We observed that X2KO cells showed decreased cell proliferation and anchorage independent growth compared to control MDA-MB-231 cells. X2KO cells also formed smaller colonies on soft agar compared with control MDA-MB-231 cells (Supplementary Figure 3).

## DISCUSSION

YAP/TAZ are regulated by both MST/LATS kinase dependent pathway and MST-independent pathway. These two pathways converge onto LATS, indicating that the requirement of LATS is indispensable [26, 27]. Therefore, elucidating LATS regulation is crucial to understanding the different modes of YAP/TAZ regulation.

To date, the only phosphatases regulating the Hippo pathway are the PP1 and PP2A phosphatases. These phosphatases dephosphorylate MST1 and YAP/TAZ [24]. It has also been reported that PP1 can interact with LATS1 and dephosphorylate LATS1 at Ser909 [5]. However, no known phosphatase has been identified to dephosphorylate LATS1 on Thr1079. In this current study, we demonstrated that POPX2 binds several of the core Hippo kinase members including MST1, NDR1 and LATS1. As MST1 shares similar structure as PAK1 in its kinase active loop, we originally hypothesized that MST1 might be a substrate of POPX2. Nevertheless, the phosphatase assays (Figure 3A and 3B) demonstrated that phospho-Thr183 on MST1 is not a substrate of POPX2. Instead, we have identified a novel role of POPX2 as a LATS1 phosphatase. POPX2 is capable of dephosphorylating LATS1 at Thr1079 (Figure 3C–3E), which is a residue critical for LATS1 activity. However, we cannot rule out the possibility that POPX2 might regulate other MST1 phospho-sites.

POPX2 has earlier been implicated in promoting early stages of cancer metastasis [28]. The outcome of dephosphorylation of LATS1 by POPX2 could lie in the control of YAP/TAZ activity and the targets of the Hippo signaling pathway. We also observed that YAP/TAZ levels in the highly metastatic MDA-MB-231 cells increased with increasing cell density which is in contrast to previous observations with NIH3T3 and MCF10A cells [25]. The difference observed could be attributed to the highly metastatic nature of the MDA-MB-231 cell line [29]. This may further suggest that the Hippo pathway is not activated to limit cell proliferation or contact inhibition in MDA-MB-231 cells.

**Figure 6:**
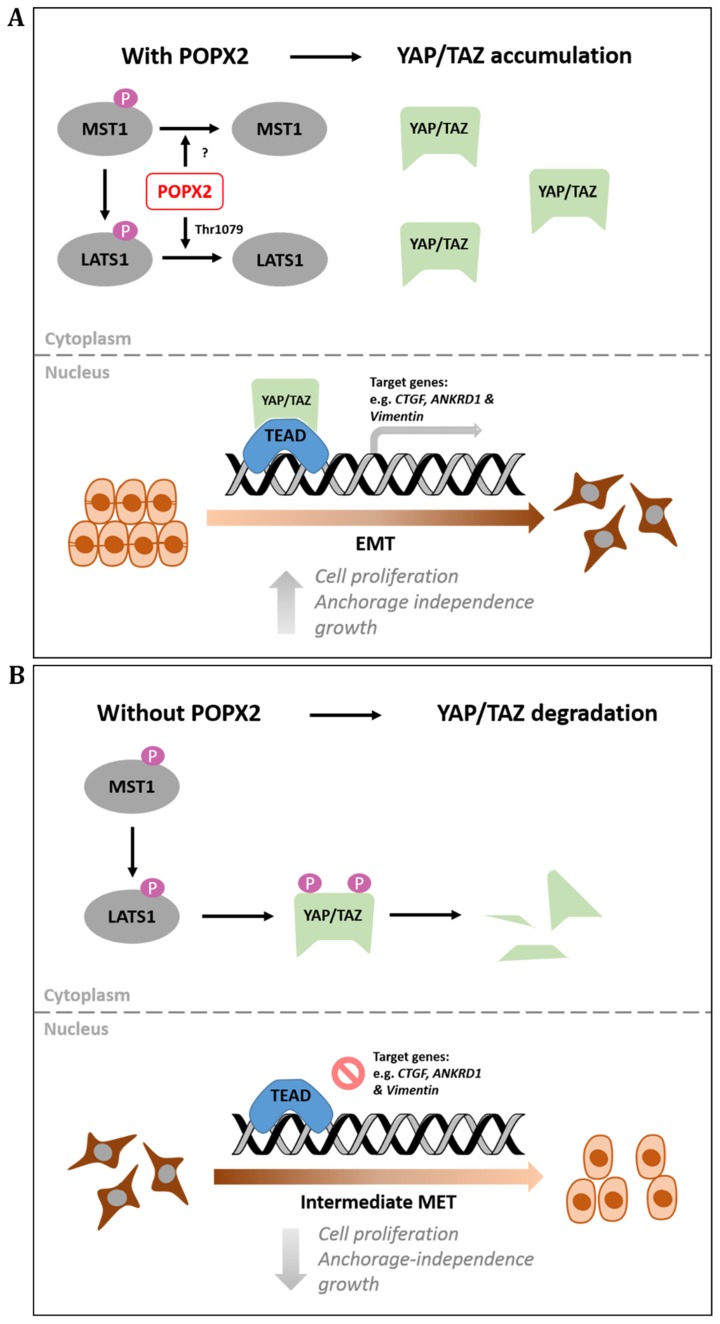
Working model for POPX2 regulation of the Hippo pathway (**A**) High levels of POPX2 lead to dephosphorylation of LATS1 on Thr1079 and inactivation of LATS1 kinase activity. Absence of LATS1 activity prevents YAP/TAZ degradation and promotes YAP/TAZ nuclear translocation. Active YAP/TAZ will then promote transcription of target genes. (**B**) When POPX2 is absent, LATS1 is phosphorylated and activated. Active LATS1 is capable of phosphorylating YAP/TAZ, which are then targeted for degradation by proteosomes.

Loss of POPX2 results in decreased YAP/TAZ protein levels (Figure 6) possibly due to increased LATS1-mediated phosphorylation and their subsequent degradation. Phosphorylation of YAP at Ser127 leads to its sequestration and retention in the cytoplasm by 14-3-3. Further phosphorylation at other residues and coordinated phosphorylation with Casein kinase 1 (CK1) lead to the ubiquitinylation of YAP by E3 ubiquitin ligase and its subsequent degradation [9]. Furthermore, loss of YAP/TAZ results in lower expression of their signature target genes, *CTGF* and *ANKRD1,* suggesting lower YAP/TAZ activity as transcription co-activators (Figure 5A and 5B).

In this study, we have demonstrated the functional consequence POPX2-LATS1 interaction. POPX2 can function to oppose the tumour suppressor function of the Hippo pathway by dephosphorylating LATS1, leading to increased nuclear YAP/TAZ. Loss of POPX2, in turn, resulted in lower levels of YAP/TAZ and thus affected anchorage independent growth. Indeed, we observed fewer colonies of X2KO when grown in soft agar compared with control MDA-MB-231 cells (Figure 5D and 5E). This is consistent with removing a negative regulator of LATS1, which resulted in increased LATS1 activity and its tumour suppressor function. However, we could not directly implicate the loss of proliferation and anchorage independence growth due to the absence of POPX2 dephosphorylation on LATS1 instead it could be an indirect effect as a result of lower TAZ level that is often associated with increased LATS1 activity. Although POPX2 has other substrates such as PAK, our observations for decreased cell proliferation and decreased anchorage-independent growth for X2KO cells are likely to be PAK-independent. This is because silencing POPX2 will lead to increased PAK activity [17], which is more likely to enhance cell proliferation. Therefore, loss of POPX2 could not lead to decrease proliferation and anchorage independent growth via the PAK pathway, but a PAK-independent pathway.

Besides its role in the Hippo pathway, LATS1 is also involved in the regulation of breast cell fate [30], the preservation of genomic integrity and initiation of programmed cell death [31], cell cycle and tetraploidy prevention [32], suppression of migration [33] and regulation of stem cell differentiation [34]. Therefore, future studies will investigate the role of POPX2 as a LATS1 phosphatase beyond the Hippo pathway.

## MATERIALS AND METHODS

### Cell culture and transfection

Both MDA-MB-231 and HEK293 cells were from ATCC (HTB-26™ and CRL-3216™). MDA-MB-231 POPX2 CRISPR cells were generated by Zhang S (Koh lab). Both HEK293 and MDA-MB-231 cells were cultured in Dulbecco’s modified Eagle’s medium (DMEM) supplemented with 10% fetal bovine serum (FBS). Transfections were carried out using Lipofectamine 2000 (Thermo Fisher Scientific) according to the manufacturer’s instructions. For different cell density plating, cells were seeded equally (0.5 × 10^6^ cells in 10-cm plate) and left to grow to desired confluency (low: 20–30%, medium: 40–60% and high: 80% cell density), growth media were replaced every 3 days.

### CRISPR/cas9-mediated human PPM1F knockout in MDA-MB-231 cells

Gene coding for PPM1F (POPX2) was knocked out through the CRISPR/Cas9-mediated genome editing system. Four single guide RNA sequences were designed with an online tool (http://crispr.mit.edu/), sgRNA1- 5′-CCGGAACACTCGCCGCAAGA-3′; sgRNA2- 5′-CTTGCGGCGAGTGTTCCGGA-3′; sgRNA3- 5′-GCAAAGTAGGCGCGGTTCAC-3′; sgRNA4- 5′-GAAACATCTGGTCGGTGCGC-3′. The annealed oligos were inserted into the GeneArt^™^ CRISPR Nuclease Vector with OFP reporter (Thermo Fisher Scientific). MDA-MB-231 cells were transfected with the respective plasmid harbouring the targeting sgRNA, followed by single cell (OFP-positive cells) sorting into 96-well plates. Single cell was expanded and harvested for POPX2 knockout validation by western blot.

### Antibodies and plasmids

Antibodies specific for the following proteins were used at the indicated dilutions for western blot: POPX2 (1:1000, Koh lab); Flag (1:5000, Sigma); HA (1:2000, Sigma); Myc (1:5000, Cell Signaling Technology); LATS1 #3477, MST1 #3682, Sav #13301, MOB1 #13730, YAP/TAZ #8418, TAZ #4883 Phospho-LATS1(Thr1079) (D57D3) #8654; Phospho-MST1(Thr 183)/MST2 (Thr180) #49332 (all 1:1000, Cell Signaling Technology); Vimentin (1:1000, Abcam); GAPDH (1:5000, EMD Millipore) and Actin (1:5000, EMD Millipore). Antibodies specific for the following proteins were used at the indicated dilutions for endogenous immunoprecipitation: PPM1F/POPX2 #56648 (1:100, Abcam); MST1 #3682 (1:100, Cell signaling) and LATS1 #8654 (1:100, Cell Signaling Technology). Antibodies specific for the following proteins were used at the indicated dilutions for immunostaining: TAZ #4883, YAP/TAZ #8418 (all 1:100, Cell Signaling Technology). Rhodamine-phalloidin was used for actin fiber staining (1:100, Life Technologies).

The following plasmids were from Addgene: pPS2977-NDR1 (from Pamela Silver, Addgene plasmid # 8927), pClneoMyc human MOB1 (from Yutaka Hata, Addgene plasmid # 37024), pcDNA3 Lats1 (Nigg HS189) (from Erich Nigg, Addgene plasmid # 41156, 3XFlag pCMV5-TOPO TAZ WT (from Jeff Wrana, Addgene plasmid # 24809). Flag-YAP2 and Flag-LATS1 were gifts from Marius Sudol, National University of Singapore.

### Generation of POPX2 antibody

POPX2 antibody was raised against POPX2 N-terminal protein (aa 1 to 150) in rabbit. Anti-sera were used in this study. We have checked that POPX2 anti-sera can recognize transfected POPX2 construct and endogenous POPX2 by Western analysis [17]. Cross activity with POPX1 has not been validated. However, POPX1 (PPM1E) is only detected in brain, testis, adrenal and parathyroid glands (The Human Protein Atlas).

### Western blot

Cells were lysed with protein lysis buffer (PLB) containing 300 mM sodium chloride, 25 mM HEPES, 1 mM MgCl_2_, 1 mM EGTA, 20 mM β-glycerophosphate, 10 mM sodium fluoride, 1 mM sodium orthovanadate, 0.5% (v/v) glycerol, 0.5% (v/v) Triton X-100, and supplemented with protease inhibitor cocktail (Roche) and PhosSTOP^™^ (Sigma). Nitrocellulose membranes were used for protein transfer. Membranes were incubated with primary antibodies in either 0.5% BSA or skimmed milk in TBST (Tris Buffered Saline + 0.1% (v/v) Tween 20) overnight, following which were washed 3 times with TBST and incubated with secondary antibody conjugated with HRP in 0.5% BSA or skimmed milk in TBST for 1 hour. Membranes were then washed 3 times with TBST.

### Immunofluorescence staining

Cells were fixed with 4% paraformaldehyde for 30 minutes and washed with PBS three times for 5 minutes each. Cells were then permeabilized with PBS containing 0.1% Triton X-100 for 10 minutes. Permeabilized cells were then incubated in blocking buffer (0.5% BSA/0.1% Triton X-100) for 1 hour, and following that with the appropriate primary antibody diluted in antibody dilution buffer (0.5% BSA/0.3% Triton X-100) overnight in 4°C. After three washes with PBS for 5 minutes each, cells were incubated with Alexa Fluor 488- or 594-conjugated secondary antibodies (1:100 dilution) for 1 hour. Cells were then washed with PBS three times for 5 minutes each and mounted with DAPI. Fluorescence was observed under Zeiss Axio Observer Z1 microscope.

### Co-immunoprecipitation

Cells were grown to confluency of 80 to 90% and were lysed with immunoprecipitation lysis buffer (IPLB) containing 25 mM Tris pH 7.5, 150 mM NaCl, 0.5% Triton-X, protease inhibitor cocktail (Roche) and PhosSTOP^™^ (Sigma). For GFP/Flag-tagged protein immunoprecipitation, Glutathione sepharose beads or mouse Anti-Flag M2 beads were washed with (IPLB) three times. 1 mg of protein lysate were then added to a bead volume of 40 µl and incubated overnight at 4°C. Beads were then washed with IPLB three times and tagged proteins were eluted by adding SDS sample buffer and boiling for 5 minutes. For endogenous immunoprecipitation, cell lysates were incubated with Protein G magnetic beads (Millipore) for 2 hours (pre-clearing). The beads were removed. Primary antibody was then added to protein lysates at a concentration of 1:100 and incubated overnight at 4°C. 10 µl of Protein G magnetic beads (Millipore) is then added to the Protein-Antibody complex and incubated at 4°C for 2 hours. The Protein G magnetic beads (Millipore) were then washed with IPLB three times and protein complex were eluted by adding SDS sample buffer and boiling for 5 minutes.

### Cell proliferation assay

Cell proliferation was analyzed using tetrazolium salt 3-(4, 5-dimethylthiazol-2-yl)-2, 5-diphenyltetrazolium bromide (MTT). Briefly, 24-well plates were seeded with 5 × 10^3^ cells in 450 µl growth medium per well. After 24 hours (Day 1) and every 48 hours (Day 3/5/7) 50 µl of 10×MTT reagent (5 mg/ml) were added to the growth medium of each wells. Plates were then incubated for 4 hours at 37°C, thereafter growth medium were removed and 200 µl of DMSO were added into each well to solubilize the formazan crystals. Spectrophotometric absorbance was measured at 570 nm after 24 hours (Day 1) and every 48 hours (Day 3/5/7).

### Soft agar assay

Cells were seeded at a density of 5 × 10^3^ cells per well in 0.35% top agar over a layer of 0.5% base agar in 60 mm culture dishes. Base agar was prepared by melting 0.1 g (1%) agarose into 10 ml sterile water (filtered through 0.2 µm pore). 5 ml of the 1% Agarose was then mixed with 5 ml of 2× complete DMEM. 2 ml of the mixture was added into the 60 mm dishes and allowed to solidify in a 37°C incubator for 30 minutes before adding the top agar. Top agar was prepared by melting 0.07 g (0.7%) Agarose into 10 ml sterile water (filtered through 0.2 µm pore). 1 ml of the 0.7% Agarose was mixed with 1 ml of 2× complete DMEM containing 5 × 10^3^ cells. The 2 ml mixture was then added onto the base agar-coated 60 mm plates and allowed to solidify in a 37°C incubator for 30 minutes before adding 2 ml of DMEM to prevent desiccation of the top agar. 2 ml of fresh media were replaced every week. After 4 weeks of incubation, colonies were stained with crystal violet. Colonies ≥50 µm in diameter were counted.

### Expression and purification of GST-POPX2

Full length POPX2 was cloned into the pGEX-4T-1 vector. pGEX-POPX2 plasmids were then transformed into Rosetta competent cells. Single cell colony were then selected and expanded into 5 ml LB broth with ampicillin and grown overnight. 2 ml of overnight culture was then added into 400 ml of LB broth and incubated with shaking till its OD reaches 0.6. Upon reaching 0.6 OD, the culture was induced with 1 mM IPTG for 16 hours at 25°C. Bacterial pellets were resuspended in STE buffer (50 mM Tris-HCL pH 7.5, 150 mM NaCl, 0.5% Triton-X, 1 mM EGTA) supplemented with 0.1 mg/ml of lysozyme, 0.5 mM PMSF, 1 mM DTT, 1.5% of Sarkosyl powder and Protease inhibitor cocktail (Roche). Lysate was then sonicated at 30% amplitude for 20 sec and 10 sec break for a total of 3 minutes. Lysate was then centrifuged at 4,000 rpm for 30 mins in 4°C. Supernatant containing soluble proteins were incubated with glutathione sepharose beads for 2 hours at 4°C, rotating. The beads were washed three times with immunoprecipitation lysis buffer (IPLB) containing 25 mM Tris pH 7.5, 150 mM NaCl, 0.5% Triton-X and protease inhibitor cocktail (Roche). GST fusion proteins were eluted with 10 mM of reduced L-glutathione (Sigma) at room temperature for 30 minutes. Dialysis of eluted protein was performed using Slide-A-Lyzer Dialysis Cassette (Thermo scientific) with buffer containing 50 mM Tris pH 8, 1 mM EGTA, 1 mM PMSF and 0.1 mM DTT. Dialysis was performed overnight in 4°C, following that dialyzed protein was collected and concentrated using Amicon- centrifugal unit (10 ml) and centrifuged at 4,000 rpm for 10–15 minutes at 4°C. Protein concentration were measured and glycerol were then added to make a final concentration of 20%. Aliquots of purified proteins were then snapped freeze in liquid nitrogen and stored in –80°C for future use.

### *In vitro* phosphatase assay

HEK293T cells were transfected with FLAG-MST1 or Flag-LATS1 with Myc-MOB1B. 48 hours later, cells were treated with 50 nM Calyculin A for 1 hour. Cells were then collected and lysed in lysis buffer supplemented with protease inhibitor. The cell lysates were then incubated with 1:50 Anti-Flag M2 antibody overnight at 4°C with constant rotation. Tagged overexpressed proteins complexed with Anti-Flag M2 antibody were then pulled-down by incubating with Protein G magnetic beads (Millipore) for 2 hours at 4°C. After washing with PBS, the magnetic beads were then incubated with different amount of bacterially expressed GST-POPX2 or calf Intestinal phosphatase, CIP (New England Biolabs, Ipswich, MA) in phosphatase buffer (50 mM HEPES [pH 7.3], 10 mM MgCl_2_, 5 mM MnCl_2_, 1 mM DTT, 0.05% Triton X-100) to a final reaction volume of 25 µl. Phosphatase reactions were carried out at 30°C for 45 min on tube agitator. The reactions were stopped by adding 5 µl of 6X SDS sample buffer and boiling for 10 minutes.

### Cell fractionation

Cells were lysed with cytoplasmic extraction reagent (CER) containing 10 mM HEPES pH7.4, 10 mM KCl, 1% IGEPAL CA-630, 1 mM DTT, protease inhibitor cocktail (Roche) and PhosSTOP^™^ (Sigma). Cells were incubated in CER at 4°C for 15 minutes. Nuclear pellets were centrifuged out at 14,000 rpm (BOECO CENTRIFUGE M-24 A) for 10 min at 4°C, and cytoplasmic fraction were obtained from the supernatant. The nuclear pellets were washed with the CER before lysing with protein lysis buffer (PLB) supplemented with protease inhibitor cocktail (Roche) and PhosSTOP^™^ (Sigma). Nuclear lysates were incubated in PLB at 4°C for 15 minutes. Nuclear lysates were centrifuged at 14,000 rpm (BOECO CENTRIFUGE M-24 A) for 10 min at 4°C, nuclear fractions were obtained from the supernatants. Both fractions were analyzed by Western blot.

### Real-Time PCR (RT-PCR)

RNA extraction was performed using RNeasy Mini Kit (Qiagen). 2.5 µg of Purified RNA was reverse-transcribed to complementary DNA (cDNA) using SuperScript VILO Reaction Mix (Invitrogen). cDNA was diluted 25×. RT-PCR was performed on a StepOnePlus Real-Time PCR System (Applied Biosystems). The 20 μl PCR reaction mixture consisted of 2 μl of cDNA, 100 nM forward and reverse primers, 2× KAPA SYBR and 50× ROX (High). Cycling conditions were initial denaturation at 95°C for 5 minutes, followed by 40 cycles consisting of 95°C for 10 seconds, 60°C for 30 seconds and 72°C for 10 seconds.

The primers used for real-time RT-PCR were as follows:

**Table:** 

Genes	Forward (5′-3′)	Reverse (5′-3′)
*β-ACTIN*	ATCAAGATCATTGCTCCTCCTGAG	CTGCTTGCTGATCCACATCTG
*POPX2*	TGTTTGATGGTCACGGAGGC	TTCTCATCCTGCCGTTCTGGT
*CTGF*	AGGAGTGGGTGTGTGACGA	CCAGGCAGTTGGCTCTAATC
*ANKRD*	AGTAGAGGAACTGGTCACTGG	TGGGCTAGAAGTGTCTTCAGAT

### General statistical analysis

Experimental data are presented as the mean ± SD. Two-tailed Student’s *t* tests with unequal variance were performed using XLMiner Analysis ToolPak on Microsoft excel. Error bars represent standard deviation. Differences were considered statistically significant when *p* values were less than 0.05 (^*^p < 0.05, ^**^p < 0.01, ^***^p < 0.001). Best represented images were shown.

## CONCLUSIONS

In the current study, we have identified POPX2 as a novel LATS1 phosphatase and a negative regulator of the Hippo pathway. We proposed that POPX2 is required for the stabilization of YAP/TAZ possibly through its regulation of LATS1 (Figure 6). The role of POPX2 as a regulator of the Hippo pathway supports previous findings that implicate its role in promoting cancer metastasis. Since POPX2 also dephosphorylates other kinases such as PAK, CaMKII and TAK1, the context and balance of these activities will likely also influence the overall outcome of POPX2’s role in cancer metastasis.

The most crucial stage to contain and restrict tumor growth is that prior to tumour cells becoming invasive. At this stage, tumor cells have yet to acquire the invasive capability that could foster their metastatic spread to distant organ. Dysregulation of the Hippo pathway and thus constitutive activation of YAP/TAZ serve as monumental point in tumor growth enabling them to overcome cell contact inhibition and anoikis [10]. The levels of POPX2 have been found to be correlated with invasiveness of breast cancer cells [17, 28]. Here, we observed that POPX2 is required for YAP/TAZ stabilization and activity. Therefore, targeting POPX2 during early stages of cancer could potentially help mitigate both the spread and growth of the cancer cells.

## SUPPLEMENTARY MATERIALS FIGURES


