# Correction: POPX2 is a novel LATS phosphatase that regulates the Hippo pathway

**DOI:** 10.18632/oncotarget.27255

**Published:** 2019-10-08

**Authors:** Muhammad Bakhait Rahmat, Songjing Zhang, Cheng-Gee Koh

**Affiliations:** ^1^ Interdisciplinary Graduate School, Nanyang Technological University, Singapore; ^2^ School of Biological Sciences, Nanyang Technological University, Singapore

**This article has been corrected:** In Figure 5E, the pictures for X2KO bottom left and bottom right were mistakenly taken from the same assay dish. A revised Figure 5 using the original raw data is shown below. The authors declare that these corrections do not change the results or conclusions of this paper.

Original article: Oncotarget. 2019; 10:1525–1538. 1525-1538. https://doi.org/10.18632/oncotarget.26689

**Figure 5:**
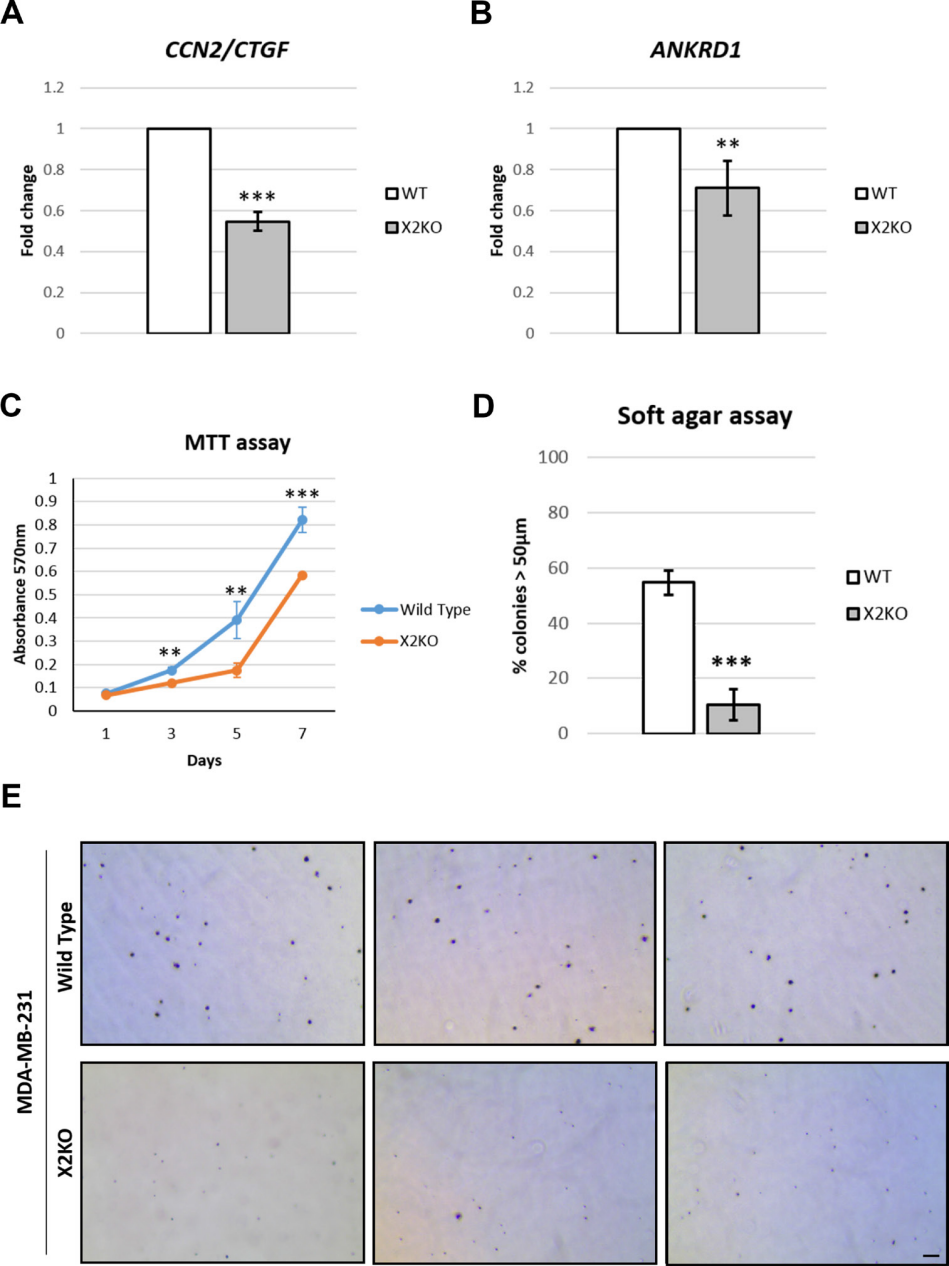
Loss of POPX2 reduces TAZ target gene expression and growth. MDA-MB-231 wild type and X2KO cells were grown to high cell density. Cells were harvested and the RNA collected were converted to cDNA and subjected to RT-PCR to quantify the amount of (**A**) *CTGF *and (**B**) *ANKRD1* transcript levels. (**C**) Cell proliferation were quantified using MTT assays on Day 1, 3, 5 and 7. Three independent experiments were performed. Error bars represent standard deviation. Student *t*-test was performed to determine statistical significance. ^**^ *p* < 0.01, ^***^ *p* < 0.001. (**D**) Cells were grown in 3D soft agar assay for up to 28 days. The numbers of total colonies and colonies with diameter more than 50 μm were counted. Three independent experiments were performed. Error bars represent standard deviation. Student *t*-test was performed to determine statistical significance. (**E**) Representative bright field images of crystal violet stained cell colonies in soft agar assay. Scale bar: 200 μm.

