# Supplementary material for: POPX2 is a novel LATS phosphatase that regulates the Hippo pathway
Source: Oncotarget. 2019 Feb 19;10(15):1525–38. doi: 10.18632/oncotarget.26689 (PMC6407677; doi:10.18632/oncotarget.26689)
Supplement: Supplementary file 1 [file oncotarget-10-1525-s001.pdf]

POPX2 is a novel LATS phosphatase that regulates the Hippo pathway

SUPPLEMENTARY MATERIALS

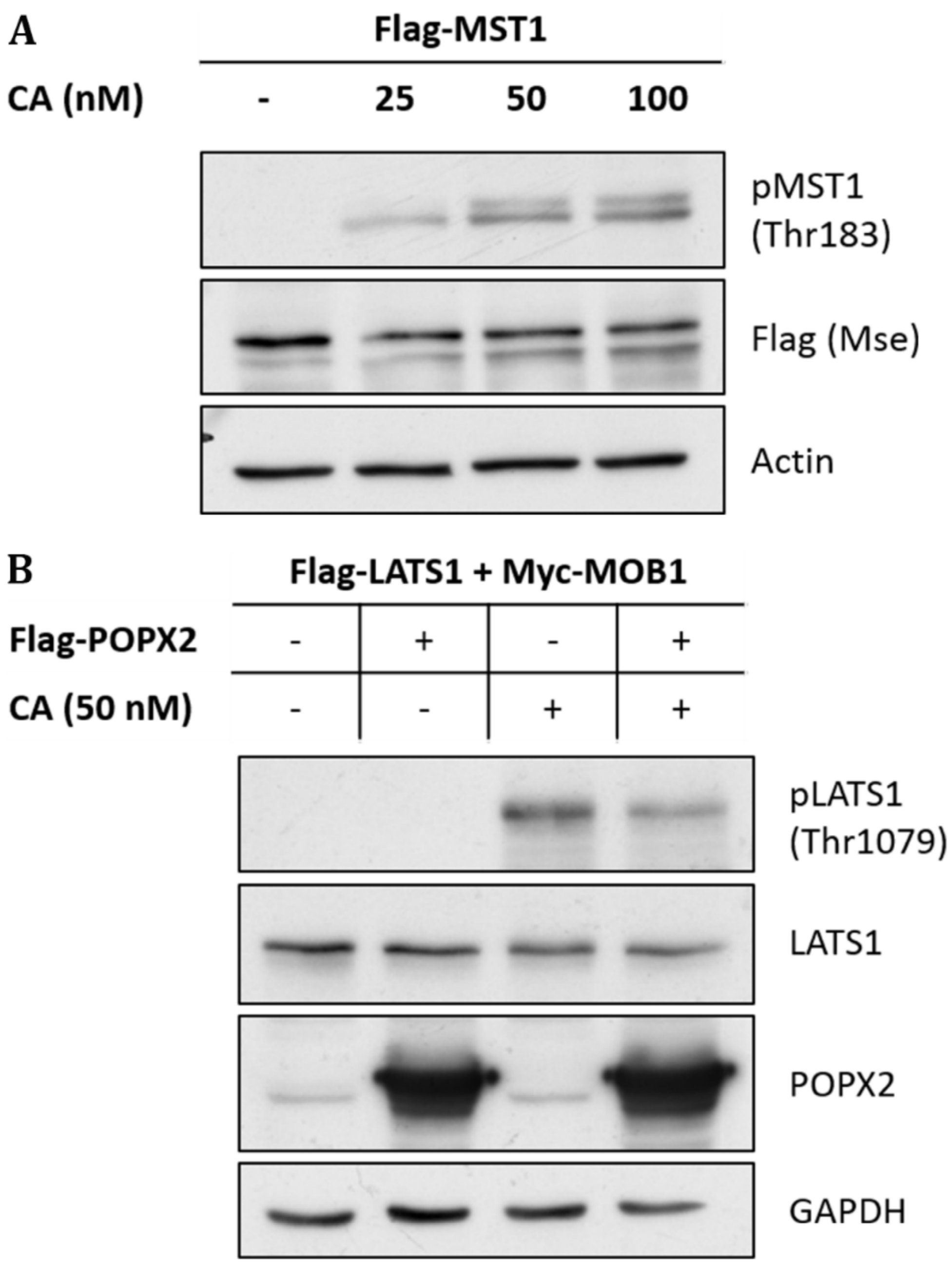

Supplementary Figure 1: (**A**) MST1 activation using Calyculin A (CA). Flag-MST1 was overexpressed in HEK293 cells and later treated with increasing dosage of Calyculin A (25, 50 and l00 nM) for 1 hr. Lysates were then subjected to SDS-PAGE and western analysis. Threonine-183 phosphorylated MST were detected using phospho-MST-Thr183 antibody, Flag-tag were detected using Anti-Flag Mouse (Mse) M2 antibody, and Anti-Actin antibody was used to detect Actin (loading control). (**B**) LATS1 activation using Calyculin A (CA). Flag-LATS1 was co-expressed with Myc-MOB1 either with or without Flag-POPX2 in HEK293 cells. Cells were later treated with 50 nM Calyculin A for 1 hr. Lysates were then subjected to SDS-PAGE and western analysis. Threonine-1079 phosphorylated LATS1 were detected using phospho-LATS1-Thr1079 antibody, total LATS were detected using LATS1 antibody, and total POPX2 were detected using POPX2 antibody, and Anti-GAPDH antibody was used to detect GAPDH (loading control).

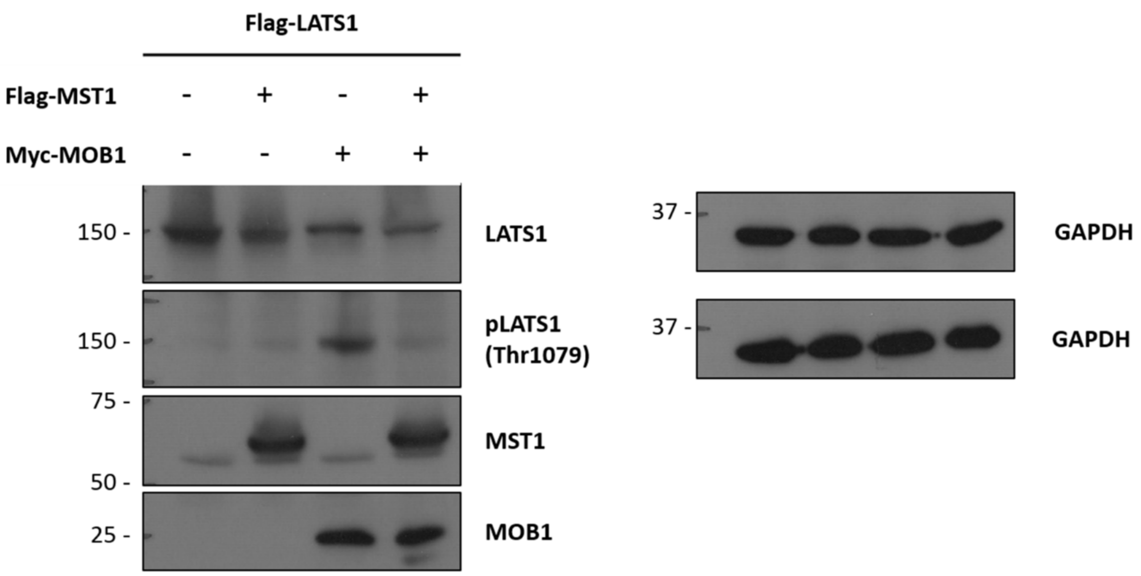

Supplementary Figure 2: LATS1 activation using MOB1. Flag-LATS1 was overexpressed in HEK293 cells alone or with Flag-MST or with Myc-MOB1 or with both. Lyastes were then subjected to SDS-PAGE and western analysis. Threonine-1079 phosphorylated LATS1 were then detected using phospho-LATS1-Thr1079 antibody, total LATS and total MST were detected using LATS1 and MST1 antibodies respectively. Total MOB1 were detected using Anti-Myc antibody. Anti-GAPDH was used to detect GAPDH (loading control). LATS1 and phospho-LATS1 were probed on different PVDF membrane from different gels but from the same sample.

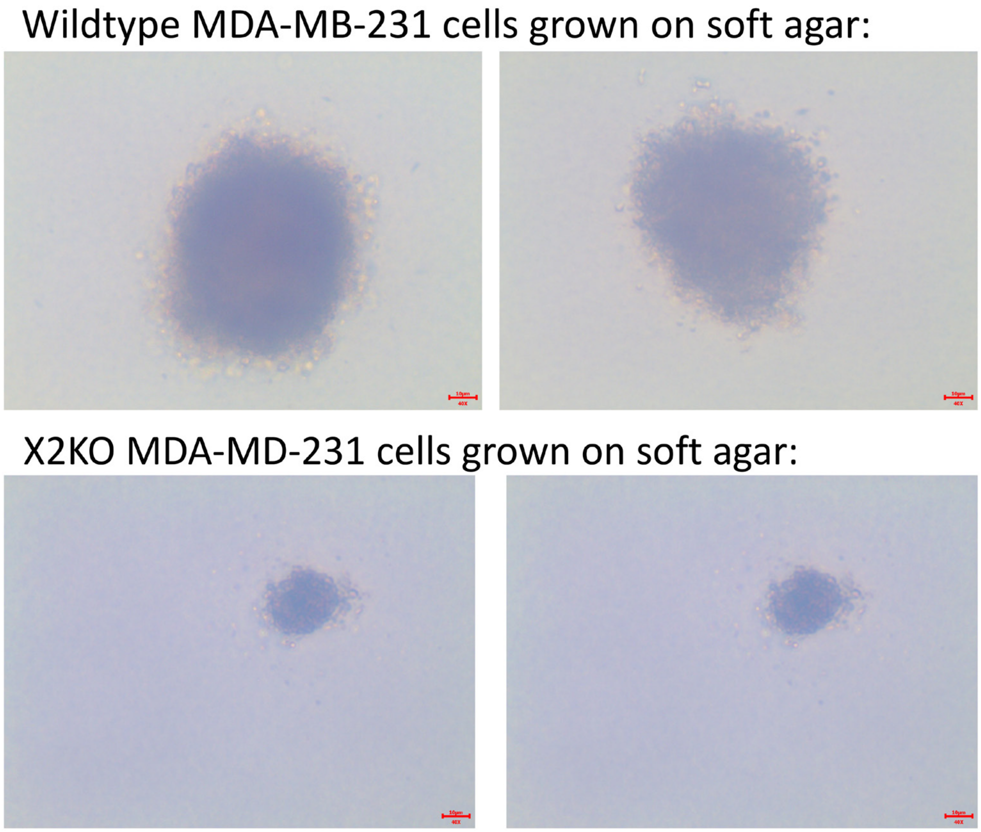

Supplementary Figure 3: Knocking out POPX2 in MDA-MB-231 cells leads to smaller colonies when grown on soft agar. After 4 weeks of incubation, the colonies were observed after staining the cells with crystal violet. Representative images of control and X2KO MDA-MB-231 cells were grown on soft agar. Scale bar: 10 µm.
